# Ultra-fast and energy-efficient sintering of ceramics by electric current concentration

**DOI:** 10.1038/srep08513

**Published:** 2015-02-17

**Authors:** E. Zapata-Solvas, D. Gómez-García, A. Domínguez-Rodríguez, R. I. Todd

**Affiliations:** 1 Instituto de Ciencia de Materiales de Sevilla, CSIC-Universidad de Sevilla, 41092 Sevilla, Spain; 2 Departamento Física de la Materia Condensada, Universidad de Sevilla, 41012 Sevilla, Spain; 3 Department of Materials, University of Oxford, OX1 3PH, UK

## Abstract

Electric current activated/assisted sintering (ECAS) techniques, such as electrical discharge sintering (EDS) or resistive sintering (RS), have been intensively investigated for longer than 50 years. In this work, a novel system including an electrically insulated graphite die for Spark Plasma Sintering (SPS) is described, which allows the sintering of any refractory ceramic material in less than 1 minute starting from room temperature with heating rates higher than 2000°C/min and an energy consumption up to 100 times lower than with SPS. The system alternates or combines direct resistive sintering (DRS) and indirect resistive sintering (IRS). Electrical insulation of the die has been achieved through the insertion of a film made of alumina fibers between the graphite die and the graphite punches, which are protected from the alumina fiber film by a graphite foil. This system localized the electric current directly through the sample (conductive materials) as in DRS and EDS, or through the thin graphite foil (non-conductive materials) as in IRS, and is the first system capable of being used under EDS or RS conditions independently combining current concentration/localization phenomena.

The sintering of ceramic materials from powders has been the subject of intense research over the last century. The use of high temperatures and long holding times in traditional techniques, such as pressureless sintering or hot pressing in conventional furnaces, has been a limiting factor for the industrial application of ceramic materials. There has therefore been a continuous quest for sintering techniques with higher energy efficiency by the scientific community. A promising approach is the use of electric current activated/assisted sintering (ECAS) techniques, in which the application of an electric current allows the sintering of ceramic materials in shorter periods of time compared to more traditional sintering techniques. There are many ECAS techniques to assist materials processing, such as powder wire discharge^1^, mechanical alloying^2^, powder consolidation^3^,^4^, chemical synthesis^5^ and solidification^6^, carried in different pieces of sintering apparatus with more than 600 patents registered^7^. ECAS processes could be divided into two main groups as proposed by Orru et al.^8^; (i) electrical discharge sintering (EDS); (ii) resistive sintering (RS). EDS is carried out for conductive materials as a high voltage electric discharge pulse, generated by a capacitor bank^4^,^9^, is forced to go through the volume of the sample located within an electrically insulating die^8^, usually made of borosilicate (BS) glass or pyrex. Softening of BS glass at high temperatures limits the use of EDS to ~800°C, which is too low for the sintering of refractory ceramic materials. Therefore, ceramists have focused on RS, which involves the application of low voltage (1–10 V) and high electric current through an electrically conducting die with the specimen under a mechanical pressure. Since its development in the early 1990s, spark plasma sintering (SPS) has been the most widely studied ECAS technique throughout the ceramic community. The industrial implementation of SPS has grown tremendously over the last few years as a consequence of the possibility of high sintering temperatures (up to ~2400°C), high heating rates (up to 600°C/min) and the application of mechanical pressures of up to ~100 MPa, which enable the sintering of ceramics in short periods of time compared with pressureless sintering or hot pressing (10–30 minutes instead of a few hours)^10^. In addition, SPS is suitable for the production of industrial scale samples with a maximum diameter of 300 mm.

In terms of energy efficiency, the power consumption of laboratory scale furnaces is around 5–10 kW for pressureless sintering, hot pressing (HP) and SPS. Recently, a transition at high temperature from electrically insulating to electrically conductive behavior has allowed the sintering of ceramics in few seconds (3–5 s)^11^ under low power dissipation conditions (~0.1 W/mm^3^) once the transition temperature is reached. This phenomenon is called flash sintering and is observed in ionic conductor and semiconductor ceramics^11^,^12^. During the flash event itself, flash sintering is more efficient than SPS but a conventional furnace is needed to reach the temperature required to induce the electrically conductive condition, thus compromising the global energy efficiency of this process. Therefore, the higher heating rate of SPS (up to 600°C/min) compared to conventional methods such as HP (50–100°C/min) and pressureless sintering (5–10°C/min) makes it the most energy efficient technique at present owing to the relatively short time over which energy is consumed. However, there remains room for improvement in the energy inefficiency during SPS processing since (i) most of the energy used is consumed in heating the graphite die rather than the specimen, (ii) the big cross section of the die produces a low resistive path by which the electric current can avoid the specimen itself. Therefore, current localization through or near to the specimen could improve the energy efficiency and shorten the process.

For this purpose, a flexible Al_2_O_3_ fiber-based film was used to electrically insulate the graphite punches from the graphite die and concentrate the electric current through the graphite punch column like in traditional EDS. Furthermore, a thin graphite foil was placed between the Al_2_O_3_ fiber-based film and the graphite punch column, as seen in figure 1a. Unlike solid ceramic insulation, an Al_2_O_3_ fiber-based film is not sensitive to thermal shock and is also thermally insulating, which minimizes thermal loss through conduction to the graphite die. In addition, the resistance of the graphite foil is ~20 times higher than the resistance of the same length of the graphite punch for a diameter of 15 mm and graphite foil of thickness 0.2 mm assuming the same resistivity for both. This high resistance of the foil ensures that the current goes through the sample for conductive materials. With electrically insulating materials, the current goes through the graphite foil, which then acts as a highly-resistive heating element, localizing the electrical heat dissipation next to the specimen. Moreover, an Al_2_O_3_ fiber-based film could be placed between the specimen and graphite punches to force the current to go through the graphite foil independently of electrical nature of the specimen, as observed in figure 1b. In addition, a piece of graphite foil is placed between the sample and the Al_2_O_3_ fiber-based film to avoid a chemical reaction during high temperature sintering as well as to improve thermal transport during ultra-fast processes in case of sintering materials with low thermal conductivity, such as Al_2_O_3_. This working mode is called forced resistive sintering (FRS) here.

The direct current mode was demonstrated using ZrB_2_, MoSi_2_ and ZrB_2_/20 vol. % MoSi_2_, all of which are electrically conductive at room temperature. These were densified by DRS for 60 seconds at different current levels as shown in figure 2. The bulk electrical resistivities of ZrB_2_ and MoSi_2_ are 10^−7^ 13 and 2 × 10^−5^ Ωm^14^, respectively. Assuming a resistivity of 10^−5^ Ωm for the graphite foil, the foil was therefore approximately 2000 and 10 times more resistive than bulk ZrB_2_ and MoSi_2_ respectively, which ensured that most of the current went through the specimen in these experiments. Figure 2 shows that the relative density increased with current level, except for ZrB_2_, which exhibited a density reduction from 91.6% to 87.9% when the current increased from 1100 A to 1170 A. A detailed comparison of the process parameters between SPS and DRS for 91.6% and 87.9% dense ZrB_2_ is shown in figure 3. A sintering temperature of 1920°C, heating rate of 100°C/min, hold time of 5 min and a pressure of 80 MPa were selected based on a previous study^15^ and the limitations of the SPS current. Regarding the 1100 A sintered ZrB_2_, the maximum current for sintering was ~30% lower in DRS than SPS whilst the voltage is higher (Fig. 3b), indicating a higher power dissipation (Fig. 3c) all of which was concentrated on the ZrB_2_ specimen itself, which resulted in a DRS energy consumption of 7% compared to SPS in this case. Note that the temperature indicated in Fig. 3a is a significant underestimate of the specimen temperature for DRS because it is measured on the unheated die rather than the specimen.

The relative density of the ZrB_2_ was 95.3% after SPS sintering. This relatively low value is mainly related to ZrO_2_ formation during sintering and residual porosity, which is related to B_2_O_3_ volatilization during sintering, as the density of ZrO_2_ (from 5.68 for cubic phase and 6.09 g/cm^3^ for tetragonal phase) is lower than for ZrB_2_ (6.12 g/cm^3^). This is difficult to avoid because ZrB_2_ powders contain ~1 wt. % of O impurity which transforms to ~3 vol. % of ZrO_2_ supposing that it completely reacts during sintering as follows:
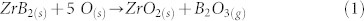
SiC and MoSi_2_ are used as sintering additives to minimize ZrO_2_ formation during sintering and fabricate a pore free ZrB_2_-based composite^15^,^16^. Although the mean density of the best DRS specimen was lower (91.6%), the density in the centre of the specimen was 95.6%, similar to that achieved with SPS. The lower mean density was caused by the lower density outer ring which can be observed in Fig. 4a. This could be removed by increasing the current (Fig. 4b) but in this case the mean density was reduced to 87.9% as a consequence of partial melting during sintering in one of the specimen faces (Fig. 4c), which was squeezed out leaving residual porosity. It should also be noted that the DRS was conducted with a lower pressure (14 MPa) than the SPS (80 MPa) in this work. Much higher densities and shorter sintering processes could presumably be achieved with the new technique by using higher pressures, which could potentially be of great help to fabricate nanostructured materials as the combination of fast heating and high pressure are key parameters to fabricate nanostructured materials^17^,^18^.

The rings formed during DRS (Figs. 4a and b) are thought to result from the thermal gradients in DRS as the temperature is reduced near the edge by conduction to the unheated surroundings. In the centre of the sample, the ZrB_2_ grains are completely surrounded by thin regions of C particles (figure 4f). The grain boundary C becomes more equiaxed and distinct far from the centre (figure 4g) and the ZrB_2_ grains become smaller. Near the edge of the sample, discrete equiaxed C particles are visible and they no longer decorate the ZrB_2_ grain boundaries (figure 4j). Pores are observed instead of C particles in the ZrB_2_ fabricated by DRS at 1100 A and the ZrB_2_ fabricated by SPS as seen in figure 4e and 4m, respectively. However, DRSed ZrB_2_ pores contain small particles of ZrB_2_ and ZrO_2_, checked by energy dispersive X-Ray spectroscopy (EDX), while empty pores are observed in SPSed ZrB_2_. Furthermore, the grain size of DRSed ZrB_2_ is visibly smaller than SPSed ZrB_2_. Ultra-fast heating higher than 2000°C/min avoids intermediate temperature coarsening, leading to faster sintering and lower grain growth compared to SPS in spite of using lower pressure during sintering. Therefore, electric current concentration could be suitable for the sintering of nanostructured materials.

B_2_O_3_ volatilization during sintering leaves residual porosity, as observed in figure 4e and 4m. C phase is observed at grain boundaries in figure 4f and 4f′ and their corresponding C maps in figures 4h and 4i. In addition, O maps are observed in figure 4 k and l, which show high O brightness in some boundaries between C and ZrB_2_. Figure 4f′ shows a microstructure after polishing and removing the melting track from the sample shown in figure 4c, which is clear evidence of a ZrB_2_-C eutectic formation. Therefore, maximum temperature is characterized by melting point of ZrB_2_-C eutectic, which is 2390°C for ~33 mol% C-ZrB_2_^19^ and indicate that gradient temperature in DRS mode is ~800°C. Furthermore, eutectic formation limits the maximum temperature for ZrB_2_ strength testing with graphite fixtures to ~2300°C^20^ and ZrB_2_ sintering using a graphite mold. Whether the C precipitates at grain boundaries or is vapor deposited is characterized by the occurrence of C sublimation.

Maximum temperature for ZrB_2_ sintered at 1170 A is ~2400°C that has a vapor pressure for C sublimation of 0.1 Pa^21^, which is lower than 3 Pa of pressure maintained during experiments. Therefore, all carbon observed in figure 4 is formed through the diffusion of C from the graphite film and punches over the ZrB_2_ lattice and precipitation at ZrB_2_ grain boundaries. Furthermore, figure 4b, c, f, f′, g and j illustrates a material produced under out of equilibrium conditions. Nonetheless, it points out the potential of DRS for producing C-MeB_2_ composites (Me = Ta, Zr, Hf), which could be of great technological interest for the scientific community as for example the bonding and impregnation between C and MeB_2_ is a current challenge to minimize ablation during hypersonic re-entries^22^. However, the current/pressure profile needs to be optimized for this purpose.

C deposition in ZrB_2_ pores is likely to be characterized by a voltage/resistivity reduction in figure 3b. ZrB_2_-C eutectic formation also accounts for relative density drop observed in figure 2. Moreover, the observation of grain boundaries revealed the existence of Zr-oxycarbides (figure 4n) as a consequence of the reaction between C and ZrO_2_ confirmed by EDX (figure 4o)^23^. Zr-oxycarbides particles were observed just in few grain boundaries as ZrO_2_ is a residual phase (1 wt.% of O impurities). Recently, it has been reported an onset of 1600° C for the generation of Zr-oxycarbides with different C/O ratio in ZrB_2_-based composites. In the cases of MoSi_2_ and ZrB_2_/20 vol. % MoSi_2_, the density and microstructure was more homogeneous throughout the sample (figure 4d), indicating that homogeneity could be controlled under DRS. The SPS temperatures to fully sinter MoSi_2_, ZrB_2_/20 vol. % MoSi_2_ and ZrB_2_ are 1300°C^24^, 1750°C^16^ and 1950°C^15^, respectively.

In the results above, the specimens conduct electricity but the graphite foil is an electrically conductive layer, which could conduct electricity when using an electrically insulating ceramic such as Al_2_O_3_, producing a RS process. Therefore, heat dissipation could be localized through the volume of the sample (DRS) or through the graphite foil (IRS) depending on electric nature of the sample. This is the first system to date suitable for EDS and RS, either DRS or IRS. Al_2_O_3_ was melted using an electric current as low as 350 A in ~52 seconds. This level of current is not sufficient to heat the conventional 15 mm graphite SPS die to 600°C in 2 minutes, as observed in figure 3a. The melting of the Al_2_O_3_ is characterized by a power spike in figure 5c as the voltage increases to keep the current constant as the fluid Al_2_O_3_ is extruded between the graphite punch and the graphite foil, reducing the conductive path and increasing the resistance. This example illustrates the magnitude of the energy savings of this new RS setting of the graphite die and the potential of the localized current sintering (LCS) technique. Moreover, temperature measured with the external pyrometer was only ~1100°C by the time the Al_2_O_3_ melted, which indicates the temperature gradient as a consequence of electric and thermal insulation could be as high as ~1000°C.

When a lower current of 340 A was used, melting was avoided and a 96.2% dense Al_2_O_3_ specimen was obtained after supplying this current for 60 s, as observed in figure 5a, b and c.

The FRS setting of Fig. 1b was also used with alumina. In this case, 98.1% dense Al_2_O_3_ was produced using a current of 410 A, as shown in figure 5. For a truly insulating specimen, little difference in response would be expected between the FRS setting and the IRS setting and the difference observed here indicates that at the high temperature during sintering, a significant current was passing through the alumina in the IRS setting.

Although the FRS setting was successful in sintering the specimen, the prevention of direct energy dissipation within the specimen reduced its energy efficiency compared to IRS. The FRS energy consumption was 50% higher than by IRS in case of Al_2_O_3_.

Figure 6a shows the microstructure of the dense Al_2_O_3_ fabricated by FRS. The microstructure consists of large Al_2_O_3_ grains (~3 μm) with clusters of nano-grains. This is a new microstructure for Al_2_O_3_, which was evidently obtained by an out-of-equilibrium process. This demonstrates the possibilities for making new microstructures with potentially new properties by these rapid heating methods.

To explore the relative merits of the different techniques, electrically conductive MoSi_2_ was also densified by FRS. Dense MoSi_2_ could be fabricated by FRS using 410 A, which is half of the current required by DRS. Figure 6b shows the microstructure of dense MoSi_2_ fabricated by DRS, in which dislocations are visible in MoSi_2_ suggesting the presence of plastic deformation during densification by DRS.

It is clear that with suitable optimization, dense specimens can be made with either the DRS/IRS or the FRS setting. There are three main considerations in optimizing these. The first is the uniformity and extent of heating. It was evident from the inhomogeneity of the structures in Fig. 1 that the DRS mode for conductive specimens can lead to severe temperature gradients because most of the heating is within the specimen and the edges are therefore in contact with relatively cold material. The success of the FRS setting in producing fully dense alumina and MoSi_2_ is at least partly because much more heating is from the graphite foil around the exterior of the specimen, conducting the heat inwards, producing a more uniform temperature profile. According to the material, the thickness of the graphite foil and therefore its resistance, and the properties of the insulating layer can potentially be balanced to give uniform temperatures and therefore improved sintering. The geometry of resistive elements could also play a role in tailoring the electric field in order to induce electric field assisted sintering (FAST) effects^25^. Similar considerations may apply to the loss of heat from the specimen to the cold graphite punches, which could be reduced with the aid of thermal insulation, including the alumina film used in this work which has a thermal conductivity of 0.2 W/(mK) while for graphite it is 81 W/(mK) according to manufacturers data.

A second optimization is for the energy efficiency. All three settings are obviously more efficient than SPS because only the specimen and its immediate surroundings are heated and because the processing times are much shorter. In the first attempt at sintering ZrB_2_ above, for instance, the improvement was by more than an order of magnitude but systematic balancing of the heating and optimization of the processing time could lead to even more dramatic reductions in energy consumption.

The third optimization is related to more accurate temperature readings. Temperature readings in the outer graphite die wall are from ~800 to 1000°C lower than real sample temperatures because of; (i) the rapidity of the process and (ii) the electrical and thermal insulation provided by the Al_2_O_3_-based film. Therefore, using a pyrometer to read the temperature on the top graphite punch near the sample, which is available in bigger and more modern SPS furnaces than the one used herein, would result in a more realistic temperature value for DRS and IRS processes. However, this limitation is still present in FRS processes although reproducibility is more accurate than in pyrometer-controlled sintering processes as DRS, IRS and FRS fully depend on current profile.

The fourth optimization, and a further benefit of these methods, is in minimizing the capacity and therefore the capital expense of the equipment used for a particular size of specimen. Recently, a concern has been raised about size limitations of samples sintered by SPS due to the need of higher currents to maintain high heating rates (100°C/min) and high temperatures for industrial size components^26^. In fact, direct current SPS technology is limited to the supply of 48 kA for a maximum sample size of 300 mm. The new LCS technique can be matched to the maximum current of the machine and achieve large specimens, more efficiently with smaller and less expensive power sources. The production of large samples would also benefit from the absolute reduction of energy used and the tailoring of thermal homogeneity in the other two areas of optimization.

In summary, the design of a novel electrically heated hot pressing system in which the die is electrically insulated from the rest of the system is described here. A current is passed through the rams and can either bypass the specimen through a graphite foil or pass through the specimen, according to the conductivity of the specimen and whether it is electrically insulated from the rams or not. The electrically insulated die is structurally equivalent to SPS die but the system is electrically equivalent to a micron sized die, which dissipates heat more locally and more quickly than a conventional SPS graphite die. This localized current sintering combines and improves on the advantages of existing techniques and enables the sintering of advanced materials and refractory ceramics by EDS and RS, either DRS/IRS or FRS, in less than 60 seconds from room temperature. The minimum contact pressure (14 MPa) was supplied in this study to demonstrate the success of this technique under low pressures but further improvements in densification could be produced by the use of higher pressure. Constant current profile was used to fabricate dense ceramics in 60 seconds. However, the combination of higher pressures up to 100 MPa in addition to optimization of heating/current profile during sintering could potentially lead to fabrication of nanostructured materials. Further advantages are energy savings of 1–2 orders of magnitude compared with SPS and the potential to produce novel microstructures owing to the high heating rate in LCS that is potentially a technique to fabricate low-cost, out of equilibrium materials, which is one of the challenges for the international ceramic community over the coming years^27^.

## Methods

1 mm thick Al_2_O_3_ fiber-based film (Morgan Thermal Ceramics, Kaowool 1600) was used to electrically insulate a graphite die for a specimen diameter of 15 mm with the help of a 0.2 mm thick graphite foil (Mersen Iberica, SPS grade). The ceramic powders used were as follows; (i) ZrB_2_ (H.C. Starck, grade B), (ii) MoSi_2_ (Sigma Aldrich, 243647), (iii) Al_2_O_3_ (Sumitomo, AKP30). A Spark Plasma Sintering furnace (Fuji Electronic Industrial Co., Dr. Sinter SPS-515S) limited to 1400 A was used for current concentration experiments. Constant pulsed DC electric current under 12:2 configuration was supplied during 60 seconds and then the SPS was shut down. However, controlled cooling was carried out in IRS for Al_2_O_3_ to avoid mechanical failure by thermal shock during cooling, reducing the current linearly in 15 minutes. The minimum contact pressure of 2.5 kN (~14 MPa for 15 mm diameter cross section) was used for safety reasons and to ensure contact at all times. Temperature was measured in-situ and recorded by an optical pyrometer in the external surface of the graphite die. Therefore, temperature differences are expected between the specimen temperature and the pyrometer reading as the Al_2_O_3_ film is a thermal insulator and the process is short enough to reduce heat loss by thermal conduction. The density after sintering was measured by the Archimedes method in distilled water. Microstructures of the bulks were characterized by TEM (Philips, CM-200), FE-SEM (Hitachi, S5200) and a FE-SEM (Hitachi, S4800) equipped with a EDX detector (Bruker, X Flash detector 4010).

## Author Contributions

E.Z.S. and R.I.T. wrote the main manuscript. All authors reviewed the manuscript. E.Z.S. designed the new graphite die, prepared all figures, performed all sintering experiments and carried out SEM studies. D.G.G. carried out TEM studies. A.D.R. and D.G.G. supervised all the experimental progress. All authors discussed and agreed the experimental approach.

## Figures and Tables

**Figure 1:**
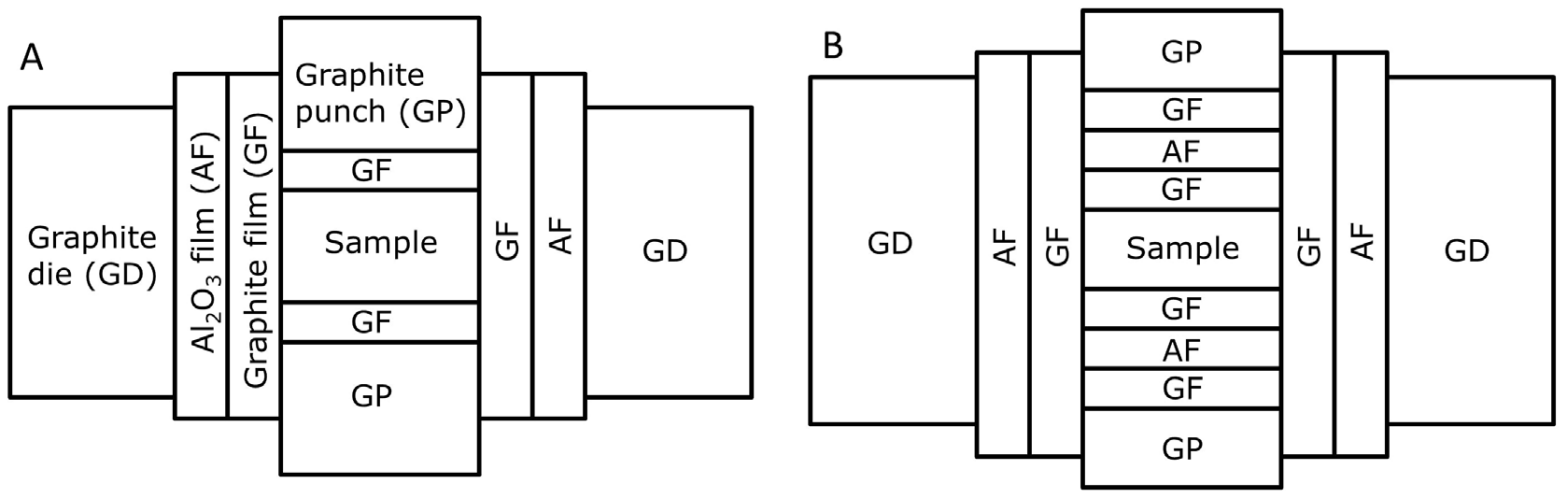
Diagram of novel settings of the graphite die for; A) DRS and IRS and B) FRS.

**Figure 2:**
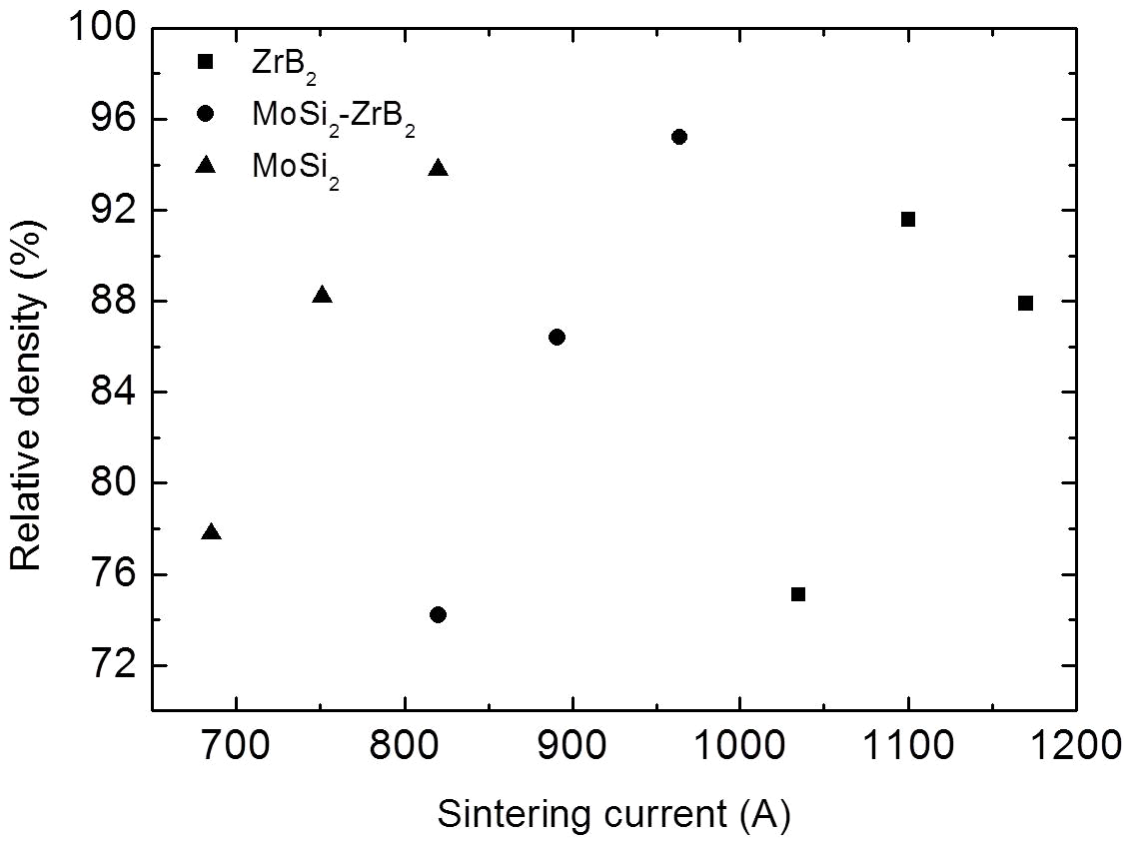
Relative density versus sintering current for DRS with current applied for 60s.

**Figure 3:**
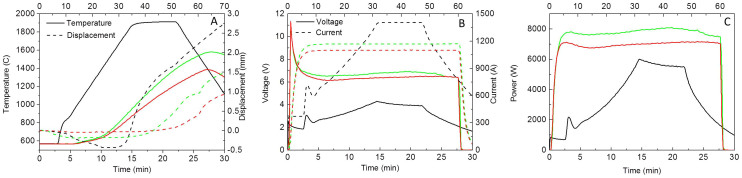
Comparison of SPS data (black lines) and DRS data at 1100 A (red lines) and 1170 A (green lines) for ZrB_2_; A) Temperature and displacement, B) Voltage and current and C) Power consumption. The time scale for SPS is on the bottom and for DRS is on the top.

**Figure 4:**
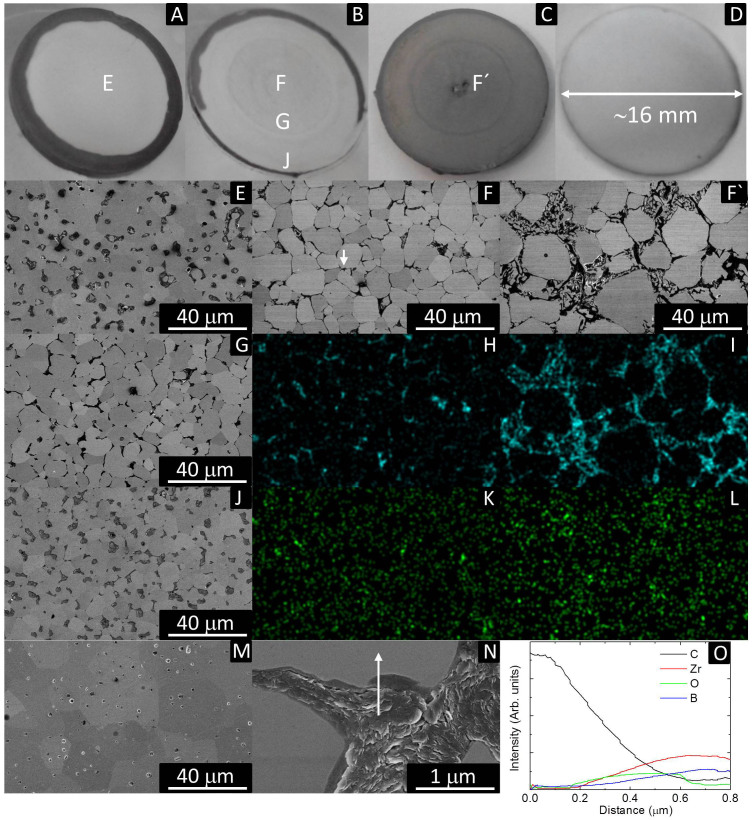
Polished cross sections of specimens sintered by DRS; A) 91.6% dense ZrB_2_ sintered at 1100 A, B) 87.9% dense ZrB_2_ sintered at 1170 A, C) other side of 87.9% dense ZrB_2_ sintered at 1170 A, D) 93.8% dense MoSi_2_ sintered at 820 A, E) Scanning electron microscopy (SEM) micrograph of area E in figure 4 A), F) SEM micrograph of area F in figure 4 B), F′) SEM micrograph of area F′ in figure 4 C), G) SEM micrograph of area g in figure 4 B), H) EDX C map of figure 4F), I) EDX C map of figure 4F′), J) SEM micrograph of area J in figure 4 B), K) EDX O map of figure 4F), L) EDX O map of figure 4F′), M) SEM micrograph of SPSed ZrB_2_, N) Magnified area marked with a white arrow in figure 4 F), O) lineal EDS indicating the concentration of C, Zr, O and B along the line in figure 4 N). Disks A and B are viewed at a certain angle to avoid direct camera reflection by the shiny mirror surface.

**Figure 5:**
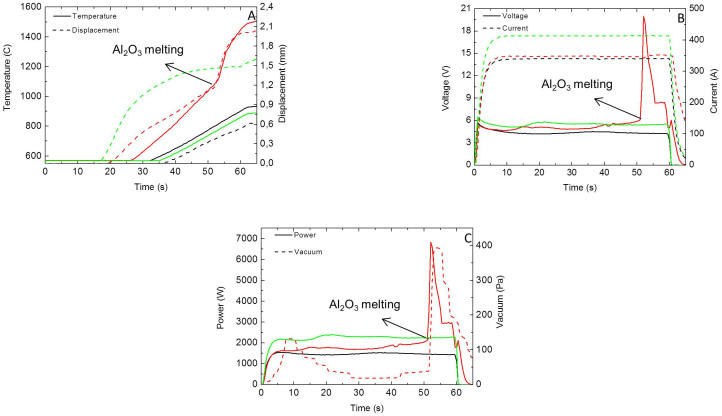
IRS data of Al_2_O_3_ sintered at 340 A (black lines) and 350 A (red lines) and FRS data of Al_2_O_3_ sintered at 410 A (green lines); A) Temperature and displacement, B) Voltage and current and C) Power consumption and vacuum.

**Figure 6:**
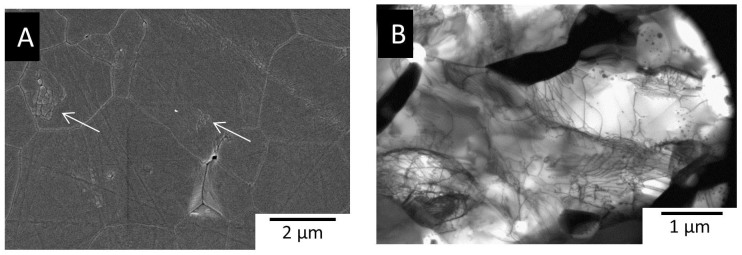
Micrographs of dense specimens; A) SEM micrograph of Al_2_O_3_, arrows indicate nanograin clusters, B) Transmission electron microscopy (TEM) micrograph of MoSi_2_.
